# The effect of lipocalin-2 (LCN2) on apoptosis: a proteomics analysis study in an LCN2 deficient mouse model

**DOI:** 10.1186/s12864-021-08211-y

**Published:** 2021-12-13

**Authors:** Dongming Wu, Xiaopeng Wang, Ye Han, Yayun Wang

**Affiliations:** Department of Emergency, Qingdao Hospital of Traditional Chinese Medicine (Qingdao Hiser Hospital), No. 4, Renmin Road, Shibei District, Qingdao, 266033 Shandong Province China

**Keywords:** Lipocalin 2, Label-free proteomics, Apoptosis

## Abstract

**Background:**

Recent studies have shown that lipocalin-2 (LCN2) has multiple functions involved in various biological and pathological processes including energy homeostasis, cancer, inflammation, and apoptosis. We aimed to investigate the effect of LCN2 on apoptosis that influences the pathogenetic process of metabolic diseases and cancer.

**Methods:**

We performed a proteomics analysis of livers taken from LCN2-knockout mice and wild type mice by using label-free LC-MS/MS quantitative proteomics.

**Results:**

Proteomic analysis revealed that there were 132 significantly differentially expressed proteins (49 upregulated and 83 downregulated) among 2140 proteins in the liver of LCN2-knockout mice compared with wild type mice. Of these, seven apoptosis-associated proteins were significantly upregulated and seven apoptosis-associated proteins downregulated.

**Conclusion:**

Proteomics demonstrated that there were seven upregulated and seven downregulated apoptosis-associated proteins in liver of LCN2-knockout mice. It is important to clarify the effect of LCN2 on apoptosis that might contribute to the pathogenesis of insulin resistance, cancer, and various nervous system diseases.

**Supplementary Information:**

The online version contains supplementary material available at 10.1186/s12864-021-08211-y.

## Introduction

Lipocalin-2 (LCN2), also known as neutrophil gelatinase-associated lipocalin, is initially an iron-trafficking protein involved in iron metabolism, innate immune function, and antibacterial infection [1–5]. LCN2 is a secreted glycoprotein belonging to the lipocalin superfamily and widely expressed in the bone marrow, gall bladder, colon, urinary bladder, stomach, and liver [1, 6–8]. Human LCN2 protein encoded by *LCN2* gene is a 23 kDa protein and has two isoforms by alternative splicing leading to a six amino acid difference in the C-terminus of the protein sequence [9].

Recently, accumulating evidence has demonstrated that the LCN2 protein is implicated in a wide range of cellular and pathophysiological processes such as insulin insistence, tumor cell proliferation, and apoptosis [8, 10–14]. Serum LCN2 protein levels were significantly elevated in patients with type 2 diabetes or impaired glucose tolerance as well as in obese women [13, 15]. Furthermore, LCN2-knockout (LCN2-KO) mice represent a metabolic phenotype of reduced fasting glucose, lipid levels, and improved insulin sensitivity. This suggests that LCN2 plays an important role in metabolic disease [16].

LCN2 expression is upregulated in many kinds of cancers and is likely involved in multiple tumorigenic processes including tumor initiation, progression, and metastasis [12]. The upregulated LCN2 protein expression is positively associated with cancer cell migration and progression in hepatocellular cancer through the hepatocyte activating growth factor receptor/focal adhesion kinase (FAK) signaling pathway [17].

LCN2 can modulate the apoptotic process by interacting with interleukin-3 (IL-3) and IL-8 to influence tumor progression, metastasis, and prognosis in FL5.12 cells, endometrial cancer, and liver cancer [4, 18, 19]. The LCN2 expression was upregulated in injured gastric mucosa with obese patients. Interestingly, the upregulated LCN2 can exert a beneficial influence on gastric mucosa via inhibition of apoptosis and inflammatory response [8]. Moreover, LCN2-KO using CRISPR/Cas 9 gene editing technology decreases tumor cell proliferation and migration, thereby enhancing cisplatin-induced apoptosis in the human prostate cancer cell line [20].

Apoptosis is known to influence the pathogenetic process of metabolic diseases and cancer. Thus far, little is known about the underlying mechanisms of LCN2 modulation of apoptosis. To investigate the regulatory mechanism of LCN2 on apoptosis, we performed a proteomic analysis of livers taken from LCN2-KO mice and wild type mice by using label-free liquid chromatography with tandem mass spectrometry (LC-MS/MS) quantitative proteomics. The results revealed multiple apoptosis-associated proteins that were upregulated and downregulated, and these might provide new insights for further therapeutic targets for metabolic diseases and cancer.

## Materials and methods

### Animals

The LCN2-KO mouse model was established as previously described [21]. Five male LCN2-KO mice and five age-matched C57BL6/J wild type (WT) mice were housed in an SPF room of constant humidity (55%) and temperature (22 ± 2 °C) under a 12-h light-dark cycle. The 8-week-old LCN2-KO mice and WT mice were allowed ad libitum access to food and water for 6 weeks. All mice were euthanized, and their livers were rapidly excised and stored in liquid nitrogen for subsequent analysis.

The study was reviewed and approved by the Animal Care and Use Committee of Qingdao Hospital of Traditional Chinese Medicine (Qingdao, China). All the surgical procedures were performed under sodium pentobarbital anesthesia and designed to minimize suffering.

### Sample preparation

SDT buffer (4% SDS, 100 mM Tris-HCl, pH 7.6) was added to the lysed liver, and transferred to 2-mL tubes. The lysate was homogenized by an MP Fastprep-24 Automated Homogenizer (6.0 M/S, 30 s, twice) (MP Biomedicals Inc., USA). The homogenate was sonicated and boiled for 15 min. After centrifugation at 14,000 *g* for 40 min, the supernatant was filtered using a 0.22-μm filter. The filtrate was quantified using the BCA Protein Assay Kit (P0012, Beyotime) [22].

### SDS-PAGE separation

Briefly, 20 μg protein for each sample was mixed with 6× loading buffer and boiled for 5 min. The proteins were separated on 12.5% SDS-PAGE. The protein bands were visualized by Coomassie Blue R-250 staining [23].

### Filter-aided Sample Preparation (FASP digestion)

Briefly, 200 μg protein were reduced with 50 mM DTT for 30 min at 56 °C. Then, the detergent, DTT, and other low-molecular-weight components were removed using UA buffer (8 M Urea, 150 mM Tris-HCl pH 8.5) by repeated ultrafiltration (Sartorius, 30 kD). Then, 100 μl iodoacetamide (100 mM IAA in UA buffer) was added to block the reduced cysteine residues, and the samples were incubated for 30 min in darkness. The filters were washed with 100 μl UA buffer three times and then 100 μl 25 mM NH4HCO3 buffer twice. Finally, the protein suspensions were digested with 4 μg trypsin (Promega) in 40 μl 25 mM NH4HCO3 buffer overnight at 37 °C. The resulting peptides were collected as a filtrate [24].

### Label-free LC–MS/MS

Samples were analyzed on a nanoElute (Bruker, Bremen, Germany) coupled with a timsTOF Pro (Bruker, Bremen, Germany) and equipped with a CaptiveSpray source. Peptides were separated on a 25 cm × 75 μm analytical column and 1.6-μm C18 beads with a packed emitter tip (IonOpticks, Australia). The column temperature was maintained at 50 °C using an integrated column oven (Sonation GmbH, Germany). The column was equilibrated using four column volumes before loading the sample in 100% buffer A (99.9% Milli-Q water, 0.1% FA); both steps were performed at 800 bar. Samples were separated at 300 nL/min using a linear gradient from 2 to 25% buffer B (99.9% ACN, 0.1% FA) over 90 min before ramping to 37% buffer B (10 min) and 80% buffer B (10 min) and was then sustained for 10 min (total separation method time: 120 min) [25].

The timsTOF Pro (Bruker, Bremen, Germany) was operated in parallel accumulation–serial fragmentation (PASEF) mode. Mass range, 100–1700 m/z (1/K0 Start 0.6 V·s/cm2 and End 1.6 V·s/cm2); ramp time, 100 ms; lock duty cycle, 100%; capillary voltage, 1500 V; dry gas, 3 L/min; dry temp, 180 °C; PASEF settings, 10 MS/MS scans (total cycle time = 1.16 s), charge range = 0–5, active exclusion = 0.4 min; scheduling target intensity, 20,000; intensity threshold, 2500; and CID collision energy, 42 eV.

### Data analysis

The MS data were analyzed using MaxQuant software version 1.6.14.0 [26]. An initial search was set at a precursor mass window of 6 ppm. MS data were searched against the Uniprot_MusMusculus_17027_20200226 database. The search followed an enzymatic cleavage rule of Trypsin/P and allowed a maximum of two missed cleavage sites and a mass tolerance of 20 ppm for fragment ions. Carbamidomethylation of cysteines was defined as fixed modification, while protein N-terminal acetylation and methionine oxidation were defined as variable modifications for database searching. The cut-off of global false discovery rate (FDR) for peptide and protein identification was set to 0.01. Protein abundance was calculated on the basis of the normalized spectral protein intensity (LFQ intensity). Proteins with fold change> 2 or < 0.5 and *p* value (Student’s *t*-test) < 0.05 were considered differentially expressed proteins [27–29].

### Western blotting

Western blotting was performed as described previously [30]. For total protein extraction, mice livers were lysed by incubation with RIPA buffer containing protease inhibitor (Shenergy Biocolor Bioscience &. Technology Company, Shanghai, China). Protein concentrations were determined using the BCA method. Equal amounts of protein were separated by 10% gel SDS-PAGE and electrophoretically transferred to a polyvinylidene difluoride membrane (Millipore, Darmstadt, Germany). The membranes were probed with primary antibodies overnight at 4 °C, and incubated with the corresponding secondary antibodies. Immune complexes were measured through Enhanced Chemiluminescence Plus Detection System.

### Antibodies

Anti-Bax antibody ((sc-70,407) was purchased from Santa Cruz.

Biotechnology (Santa Cruz, CA). Anti-Deptor antibody (ab191841), anti-Stat1 antibody (ab140412), anti-β actin antibody (ab6276), and GAPDH antibody were obtained from abcam (Cambridge, UK). β-tubulin antibody was from ABclonal (Wuhan, China). Anti-Rps6ka1 Polyclonal antibody was from Absin (Shanghai, China).

### Tunel assay

Liver tissues from WT and LCN2-KO mice were formalin fixed and paraffin embedded. Apoptosis in paraffin-embedded sections was detected by terminal deoxynucleotidyl transferase (TdT)-mediated deoxyuridine triphosphate (dUTP) nick end labeling (Tunel) assay using a commercial kit (servicebio technology CO.,LTD, Wuhan, China) according to the manufacturer’s instructions. Briefly, the liver section was washed in 2 changes of xylene 15–20 min each, and followed by dehydrate in a gradient ethanol and distilled water. The section was incubated with proteinase K at 37 °C for 25 min and permeabilized. After the slices are slightly dried, buffer is added to the tissues in the circle, and the buffer is incubated at room temperature for 10 min. The tissue was incubated with appropriate amount of solution containing TdT, fluorescein (FITC)-labelled dUTP and buffer in a flat wet box for 120 min at 37 °C. The slides were washed with phosphate-buffered saline and incubated with diamidino-2-phenylindole (DAPI) solution at room temperature for 10 min. Microscopic examination and collecting images was through fluorescence microscope. DAPI emits blue and FITC emits green, respectively.

### Gene Ontology (GO) analysis

First, all protein sequences were aligned to the *Homo sapiens* database downloaded from NCBI (ncbi-blast-2.2.28 + −win32.exe), only the sequences in top 10 and E-value<=1e-3 were retained. Second, the GO term (database version: go_201504.obo) of the sequence was selected with top Bit-Score by Blast2GO [31]. Next, we completed the annotation from GO terms to proteins by Blast2GO Command Line. After the elementary annotation, InterProScan was used to search the EBI database by motifs and then, the functional information of motif to proteins was added to improve annotation. Then, further improvement of annotation and connection between GO terms was performed with ANNEX. Fisher’s exact test was used to enrich the GO terms by comparing the number of differentially expressed proteins and total proteins correlated to GO terms [32]..

### Kyoto Encyclopedia of Genes and Genomes (KEGG) pathway annotation

Pathway analysis was performed using the KEGG database. Fisher’s exact test was used to identify the significantly enriched pathways by comparing the number of differentially expressed proteins and total proteins correlated to pathways.

## Results

### Differentially expressed apoptosis-associated proteins in the liver between LCN-KO mice and WT mice

**Fig. 1:**
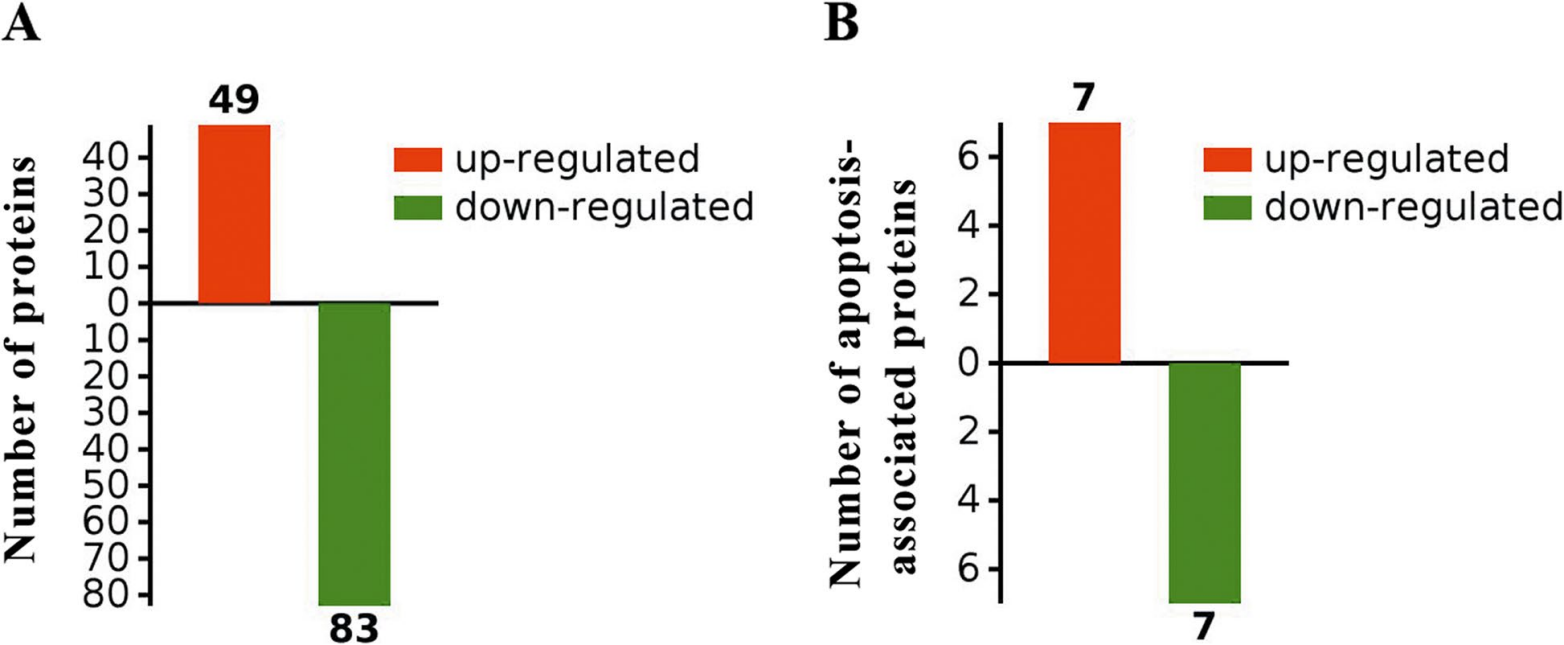
Identification of differentially expressed proteins and apoptosis-associated proteins in the liver of LCN2-KO mice compared with WT mice. **A** The differentially expressed proteins of the liver of LCN2-KO mice compared with WT mice. **B** The proteomic changes of 14 apoptosis-associated proteins from the differentially expressed proteins

To identify the proteins differentially expressed in the liver of LCN-KO mice, liver extracts from LCN2-KO and WT mice were comparatively analyzed by label-free quantitative proteomics. The proteomics analysis revealed 132 significantly differentially expressed proteins (49 upregulated and 83 downregulated [fold change≥2, *P* < 0.05]) among the 2140 proteins that had quantification information (data not shown) in the liver of LCN2-KO mice compared with WT mice (Fig. 1A). Of these, seven apoptosis-associated proteins were significantly upregulated and seven apoptosis-associated proteins downregulated (Fig. 1B). A list of 259 apoptosis-associated proteins with their fold changes and 14 differentially expressed proteins are shown in Table 1 and supplementary file.

**Table 1:** Identification of differentially expressed apoptosis-associated proteins in the liver of LCN2-KO mice compared with WT mice

Change	Protein IDs	Protein Name	Gene Name	Fold Change
up	Q07813	Apoptosis regulator BAX	Bax	5.40
up	P49710	Hematopoietic lineage cell-specific protein	Hcls1	3.37
up	Q570Y9	DEP domain-containing mTOR-interacting protein	Deptor	3.14
up	Q9D7X8	Gamma-glutamyl cyclotransferase	Ggct	2.93
up	P10107	Annexin A1	Anxa1	2.56
up	Q99M87	DnaJ homolog subfamily A member 3	Dnaja3	2.39
up	Q9JLV5	Cullin-3	Cul3	2.25
down	P42225	Signal transducer and activator of transcription 1	Stat1	0.49
down	P18653	Ribosomal protein S6 kinase alpha-1	Rps6ka1	0.47
down	Q8VDQ8	NAD-dependent protein deacetylase sirtuin-2	Sirt2	0.36
down	P61082	NEDD8-conjugating enzyme Ubc12	Ube2m	0.24
down	Q9EST5	Acidic leucine-rich nuclear phosphoprotein 32 family member B	Anp32b	0.22
down	O88738	Baculoviral IAP repeat-containing protein 6	Birc6	0.16
down	Q9JIZ0	Probable N-acetyltransferase CML1	Cml1	0.13

Note: up, upregulation; down, downregulation

### Bioinformatics analysis of differentially expressed apoptosis-associated proteins

**Fig. 2:**
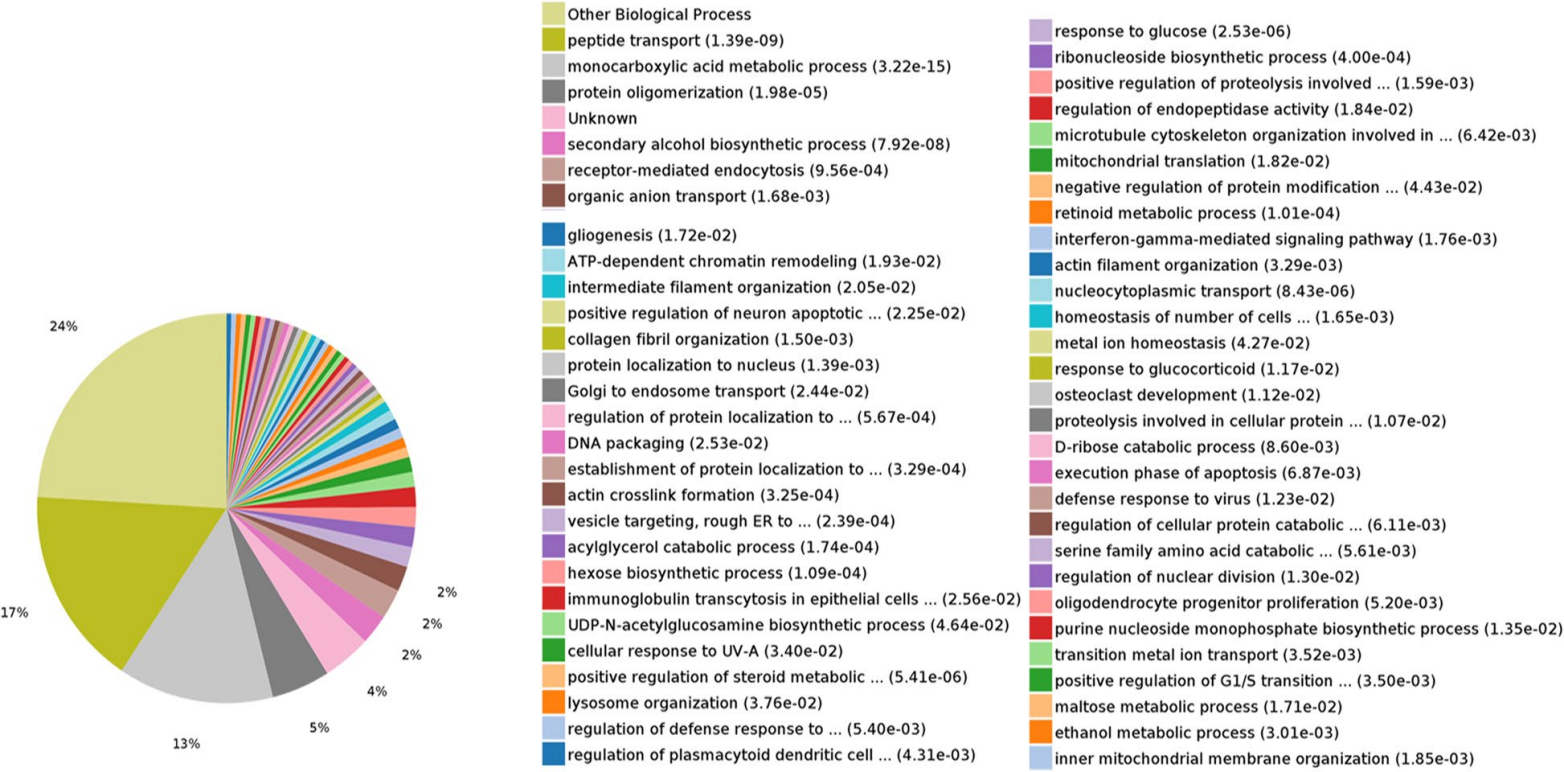
GO analysis of biological processes of identified proteins

To further investigate the effect of LCN2 on the apoptotic process, we analyzed the proteomic data using GO analysis and KEGG pathway enrichment analysis. As shown in Fig. 2, 17% of all identified differentially expressed proteins were implicated in peptide transport process, and 13% of proteins were involved in the monocarboxylic acid metabolic process. Fourteen differentially expressed proteins in the liver of LCN2-KO mice were likely associated with the regulation of the neuronal apoptotic processes.

**Fig. 3:**
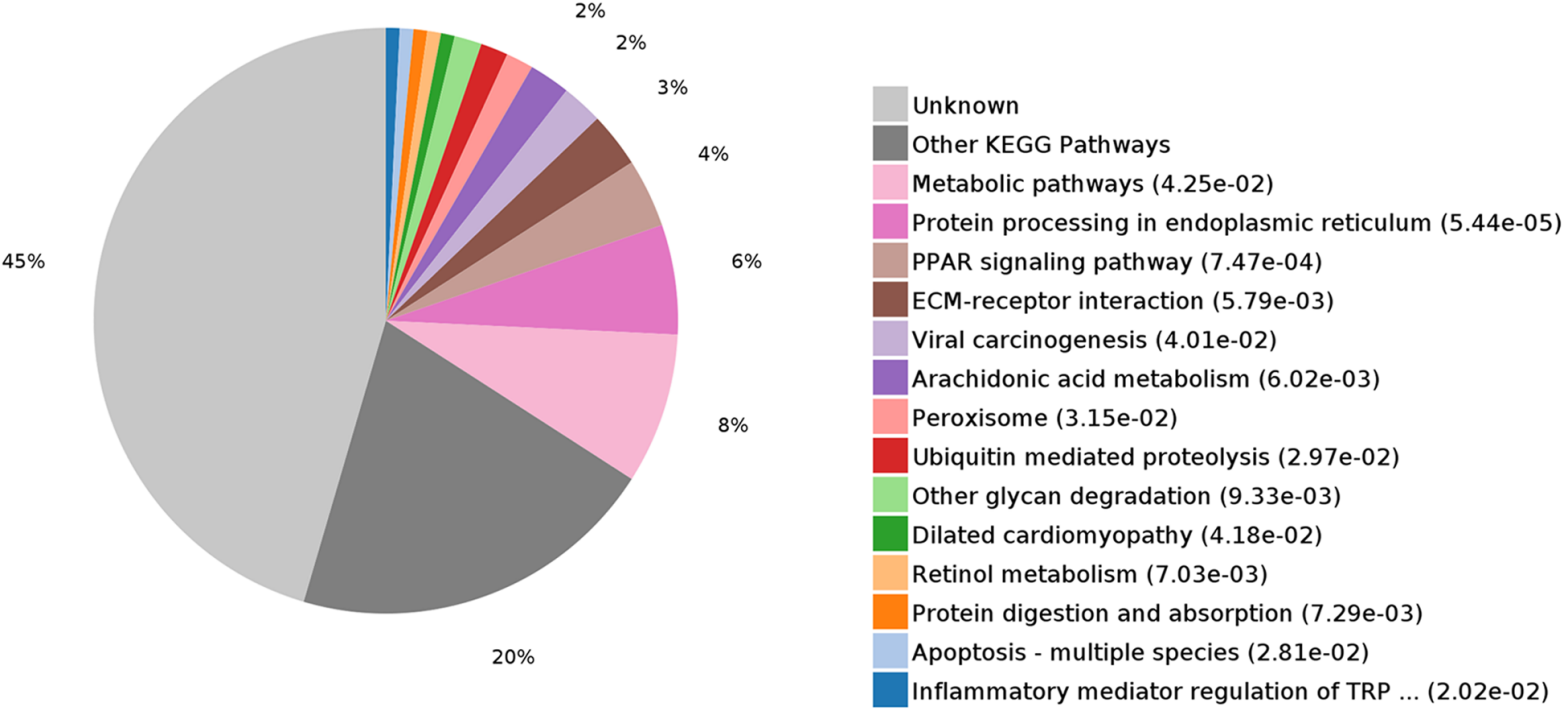
KEGG pathway analysis of differentially expressed proteins

KEGG analysis revealed that 8% differentially expressed proteins were associated with metabolic pathways; these indicated that LCN2 played an important role in metabolism. According to the above-mentioned results, several differentially expressed proteins were linked to regulating apoptotic process (Fig. 3).

**Fig. 4:**
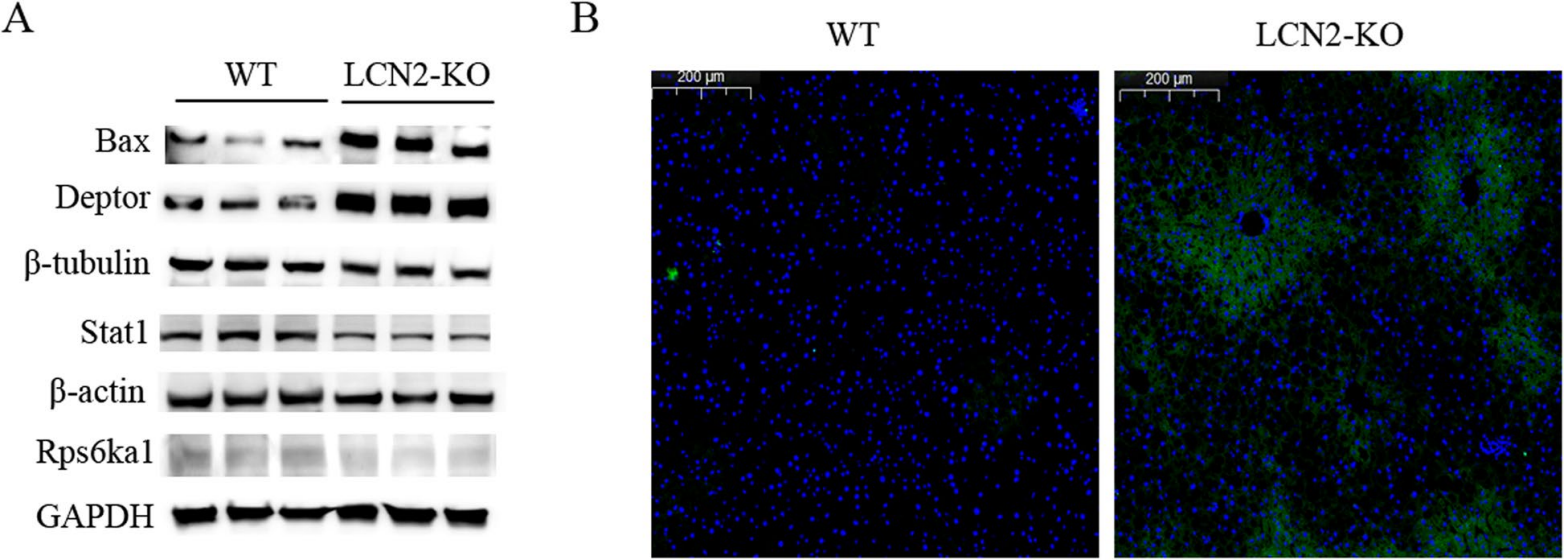
Apoptosis was evaluated using the Tunel assay, DAPI nuclear staining, and Western blotting analysis. **A**. Upregulation of Bax and Deptor protein, and downregulation of Stat1and Rps6ka1 protein in the livers of the LCN2-KO mice were determined by western blotting. Experiments were conducted in triplicates. Full-length blots/gels were presented in Supplementary file Fig. S1, S2, S3, S4, S5, S6 and S7. **B**. Representative images of the Tunel assay in the livers of the WT and LCN2-KO mice. Nucleus is blue by labeling with DAPI. Positive apoptosis cells are green. Scale bar, 200 μm. Abbreviations: Bax: apoptosis regulator BAX. Deptor: DEP domain-containing mTOR-interacting protein. Stat1: signal transducer and activator of transcription 1. Rps6ka1: Ribosomal protein S6 kinase alpha-1

**Fig. 5:**
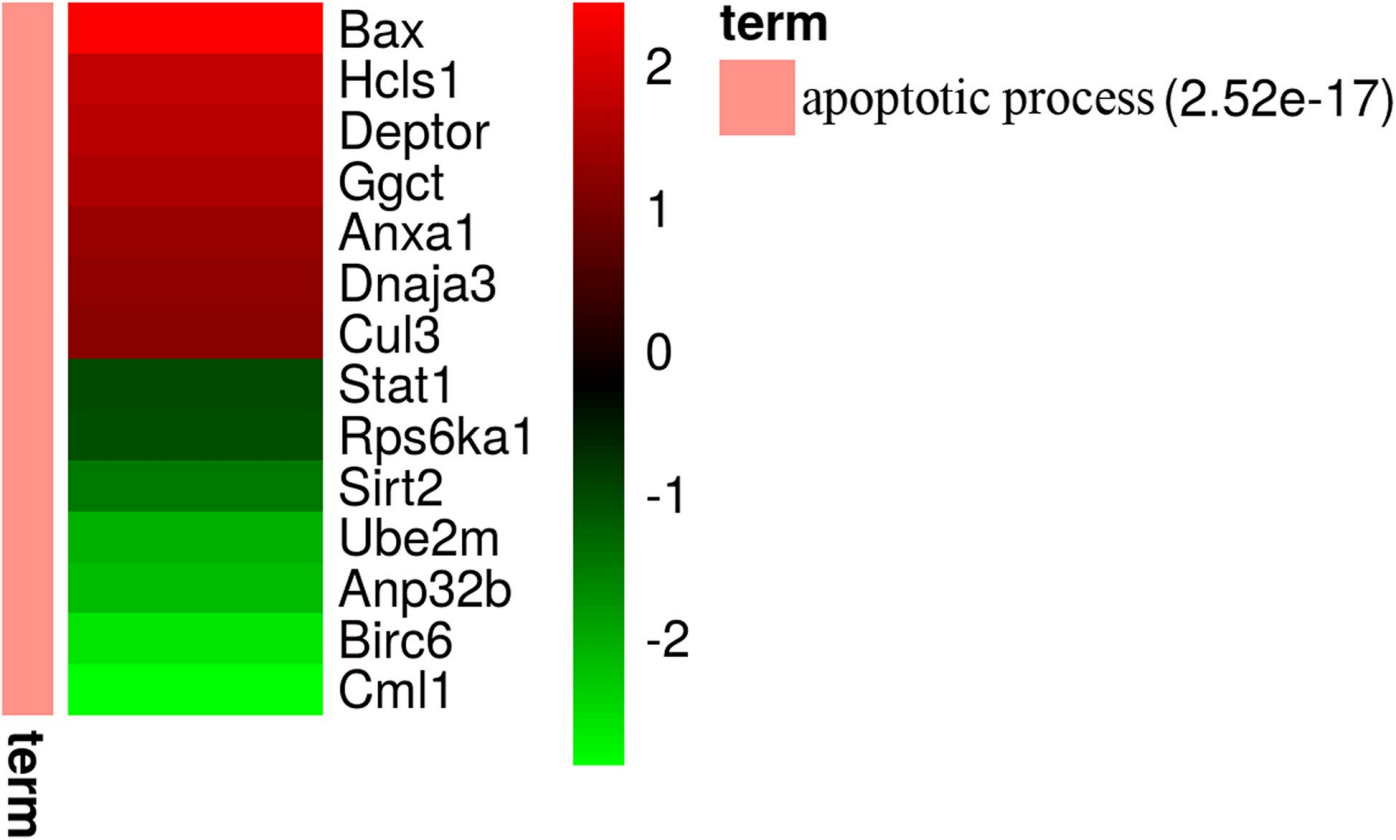
Heatmap of 14 differentially expressed proteins in apoptotic process

**Fig. 6:**
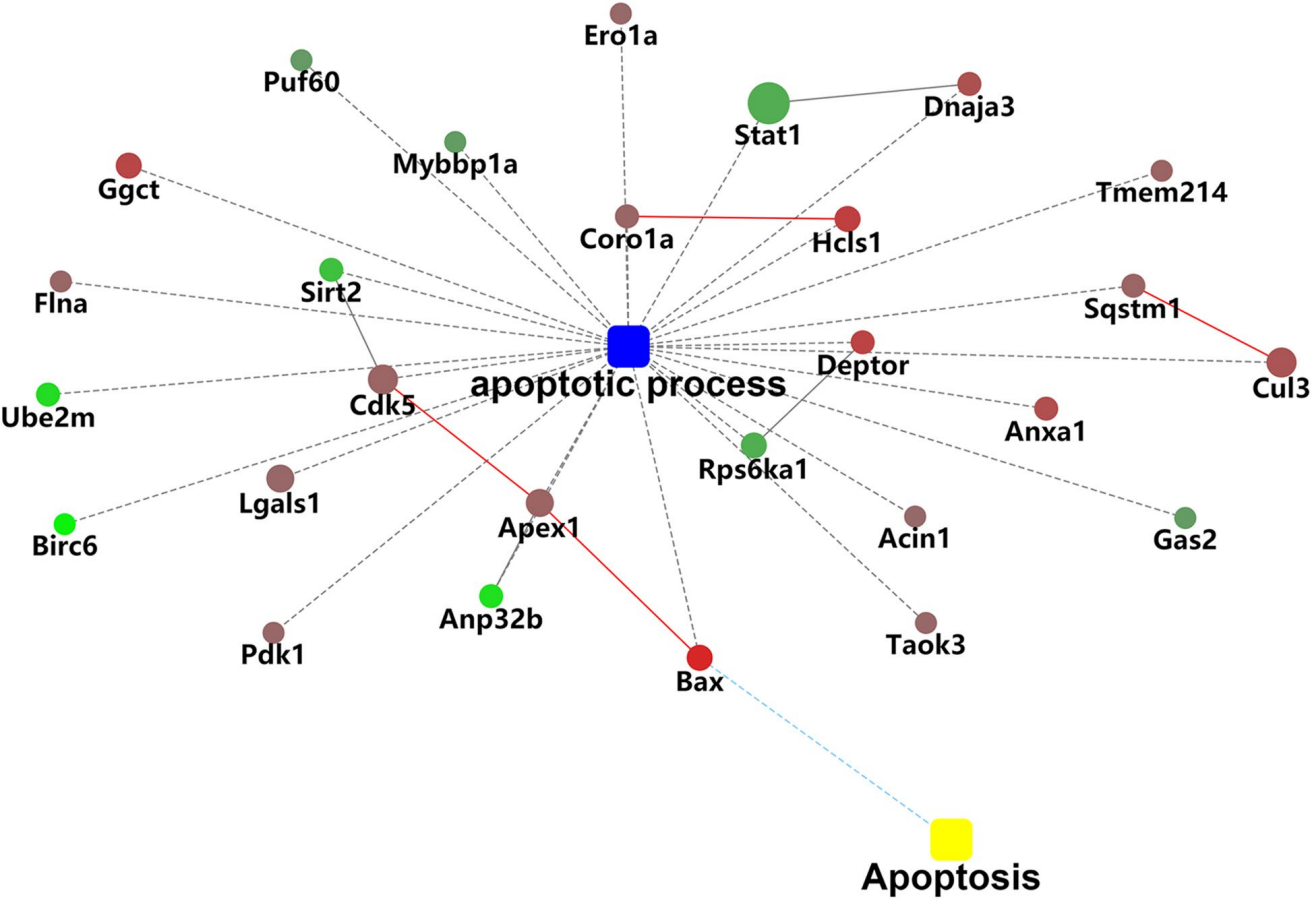
Protein–protein interaction (PPI) network analysis of differentially apoptosis-associated proteins. The following network model is generated using Cytoscape web application, based on information gained up to four levels of functional analysis: fold change of gene/protein, protein-protein interaction, biological process enrichment, andKEGG pathway enrichment (http://www.genome.jp/kegg/) [33, 34]. Circle nodes for genes/proteins and rectangle for KEGG pathway or biological process. Pathways are colored with gradient color from yellow to blue, where yellow represents smaller *P*-value and blue represents larger *P*-value. Biological processes are colored with red. In case of fold-change analysis, genes/proteins are colored in red (up-regulation) and green (down-regulation). Default confidence cut-off of 400 was used: interactions with bigger confident scores are shown as solid lines between genes/proteins; otherwise, as dashed lines

To confirm the effect of differentially expressed proteins on apoptosis in LCN2-KO mice, western blotting was used to detect the 4 significantly changed proteins, and Tunel assay was performed to evaluate the apoptotic process in the liver of LCN2-KO mice. In consistent with proteomics result (Table 1), we also observed an obvious increase of apoptosis regulator BAX (Bax) and DEP domain-containing mTOR-interacting protein (Deptor) protein level, and an obvious decrease of signal transducer and activator of transcription 1 (Stat1) and Ribosomal protein S6 kinase alpha-1 (Rps6ka1) protein level. Tunel assay suggested evidence of increased apoptosis in the liver of LCN2-KO mice (Fig. 4). We visualized 14 differentially expressed apoptosis-associated proteins using a heatmap (Fig. 5). To better understand the regulatory role of 14 differentially expressed apoptotic proteins, we further established a network of protein–protein interactions (PPIs) between the identified differentially apoptotic proteins and other possible factors (Fig. 6). Various apoptosis-associated proteins interacted with each other and formed a complicated network suggesting a complex regulatory mechanism.

## Discussion

LCN2 is primarily an iron-trafficking protein involved in the regulation of cellular iron ion homeostasis, and recent studies have shown that LCN2 had multiple functions involved in various biological and pathological processes including energy homeostasis, cancer, inflammation, and apoptosis [3, 8, 12, 19, 35].

Several studies have reported that LCN2 protein expression is upregulated in human diseases such as obesity, type 2 diabetes, and a wide range of cancers [8, 12, 15, 36]. Many studies have investigated and revealed the underlying mechanisms implicated in multiple pathways [12, 37]. Downregulation of LCN2 has been reported in oral squamous cell carcinoma [38]. Law et al. demonstrated that insulin resistance and obesity could be improved in mice with *LCN2* gene KO, suggesting LCN2 to be a risk factor, wherein the study by Guo et al. showed that insulin resistance and obesity could be potentiated in mice with *LCN2* gene KO, suggesting LCN2 to be a protective factor [16, 39]. These suggest that some contradictory findings need to be further investigated and addressed.

Apoptosis played an important role in human disease processes such as cancer, autoimmune disease, metabolic disease, and ageing [8, 40, 41]. In our study, we showed that 14 differentially expressed proteins were related with apoptotic processes in the liver of LCN2-KO mice suggesting that LCN2-induced apoptosis was critical for regulation of cellular biological processes.

Proteomics demonstrated that there were seven upregulated apoptosis-associated proteins mediated by *LCN2* in the mouse liver. A previous study has revealed that LCN2 induced apoptosis by inhibiting B-cell lymphoma 2 (Bcl-2)-associated agonist of cell death (Bad) and another Bcl-2 family member, (Bcl-X_L_), under the condition of interleukin-3 (IL-3) deprivation in FL5.12 lymphocytic cells [4]. In our study, the levels of Bad and Bcl-X_L_ proteins and that of Bcl-2-associated transcription factor 1 (Btf) and Bcl-2-like protein 13 (Bcl2-L-13) did not significantly change (supplementary file). Proteomics analysis showed that seven differentially expressed apoptosis-associated proteins were upregulated in the liver of LCN2-KO mice. Apoptosis regulator BAX (Bax) could activate caspase-3 and the apoptotic process under stress conditions involved in the regulation of apoptotic signaling pathway interaction with Bcl-2 or Bcl-X_L_ [42]. Our study showed that Bax was a pro-apoptotic protein and was significantly upregulated by 5.4-fold suggesting that LCN2 was an important regulatory molecule of apoptotic signaling pathway in the liver.

The hematopoietic lineage cell-specific protein (Hlcs1) is a substrate of the antigen receptor-coupled tyrosine kinase and plays a role in B lymphocyte cell antigen receptor (BCR)-mediated apoptosis and the regulation of tyrosine phosphorylation of signal transducer and activator of transcription (Stat) protein [43, 44]. DEP domain-containing mTOR-interacting protein (Deptor) was physiologically involved in the inhibition of mammalian target of rapamycin (mTOR) signaling pathway and induced apoptosis [45]. Gamma-glutamyl cyclotransferase (Ggct) induced apoptosis by promoting the release of cytochrome C from mitochondria [46]. Future studies should investigate the physiological significance of these three upregulated proteins in LCN2-KO mice.

The DnaJ homolog subfamily A member 3 (Dnaja3) regulates apoptotic signal transduction by influencing cytochrome C release from the mitochondria and activating caspase 3 [47]. Dnaja3 protein was upregulated in the hippocampi of Alzheimer’s disease (AD) patients and the AD mouse model, and Dnaja3 induced amyloid β42 (Aβ42) production and neuronal apoptosis, which played a crucial role in the pathogenesis of AD [48, 49]. Recently, a growing body of evidence has indicated that the LCN2 was associated with AD pathogenesis, vascular dementia, and other neurodegenerative dementias [50, 51]. There was a significant increase of Dnaja3 in the liver of LCN2-KO mice indicating a close association between LCN2 and Dnaja3. The GO analysis revealed that LCN2 was involved in positive regulation of neuronal apoptosis. However, despite no detection of LCN2 in the neurons of LCN2-KO mice, we speculate that Dnaja3 is likely an important mediator of LCN2-induced apoptosis in nervous system diseases.

Our data revealed that seven apoptosis-associated proteins were obviously decreased in the liver of LCN2-KO mice, in which Stat1 was the most drastically decreased. Stat1 mediates multiple biological processes including cell development, angiogenesis and insulin signal pathway, and stimulates caspase activity and IFN-induced apoptosis [52, 53]. Early studies have demonstrated that IFNγ can activate Stat1 and Stat1 can directly bind to the promoter of *LCN2* gene and upregulate its expression in adipocytes, which suggested that Stat1 likely played a critical role in LCN2’s apoptotic function [54, 55]. Stat1 also interacted with Dnaja3 to influence IFNγ-indued apoptosis (Fig. 6) [56]. Therefore, numerous proteins interacted with other regulators to represent different effects on biological functions such as inhibition or activation.

Ribosomal protein S6 kinase alpha-1 (Rps6ka1) is a serine/threonine-protein kinase that regulates apoptosis by phosphorylating Bad and repressing Bad function [57, 58]. Consistent with previous findings by Devireddy et al., LCN2 possibly induced apoptosis through Rps6ka1-mediated phosphorylation of Bad [4].

NAD-dependent protein deacetylase sirtuin-2 (Sirt2) deacetylates downstream target proteins to modulate diverse cellular processes such as cell cycle, apoptosis, differentiation, metabolism, and autophagy [59]. Sirt2 reduces oxidative stress-induced apoptosis by influencing the cMYC (a transcription factor) pathway in cholangiocarcinoma [60]. However, according to present literature, it has not been reported in the direct interaction of LCN2 and Sirt2.

NEDD8-conjugating enzyme Ubc12 (Ube2m) interacts with E3 ubiquitin ligase RBX1 to play a role in the positive regulation of neuron apoptotic processes and cell proliferation through neddylation modification of Cullin3 (Cul3) [61, 62]. The interaction of Ube2m and Cul3 might be associated with the LCN2-mediated regulation of neuronal apoptosis (Fig. 2). Nonetheless, the exact mechanism of LCN2-mediated apoptosis through these apoptosis proteins or interaction is still not well studied and needs further investigation.

Our study focused on the effect of LCN2 on apoptosis which is involved in the pathogenesis of insulin resistance, cancer, and nervous system diseases. Our results might provide new insights in the development of novel therapeutic targets for metabolic diseases and/or cancer. A limitation of the study was the lack of validation of the identified upregulated and downregulated apoptosis-associated proteins. Further studies are required to investigate the physiological significance of these LCN2-mediated apoptotic proteins in the liver and other tissues.

## Conclusion

Our proteomics analysis has identified seven upregulated and seven downregulated apoptosis-associated proteins in the liver of LCN2-KO mice. It is vital to clarify the effect of LCN2 protein on apoptosis as the latter likely contributes to the pathogenesis of insulin resistance, cancer, and various nervous system diseases.

## Supplementary Information

**Additional file 1: Figure S1.** Full-length blot of Bax protein expression level. **Figure**. **S2.** Full-length blot of Deptor protein expression level. **Figure**. **S3.** Full length blot of β-tubulin protein expression level. **Figure S4.** Full-length blot of Stat1 protein expression level. **Figure S5.** Full-length blot β-actin protein expression level. **Figure S6.** Full-length blot of Rps6ka1 protein expression level. **Figure S7.** Full length blot GAPDH protein expression level.

**Additional file 2.**

## Data Availability

All data generated and analyzed during our study are included in the published article and supplied in one supplementary file.
