# Supplementary material for: The effect of lipocalin-2 (LCN2) on apoptosis: a proteomics analysis study in an LCN2 deficient mouse model
Source: BMC Genomics. 2021 Dec 13;22:892. doi: 10.1186/s12864-021-08211-y (PMC8670060; doi:10.1186/s12864-021-08211-y)

Figure S1 Full-length blot of Bax protein expression level

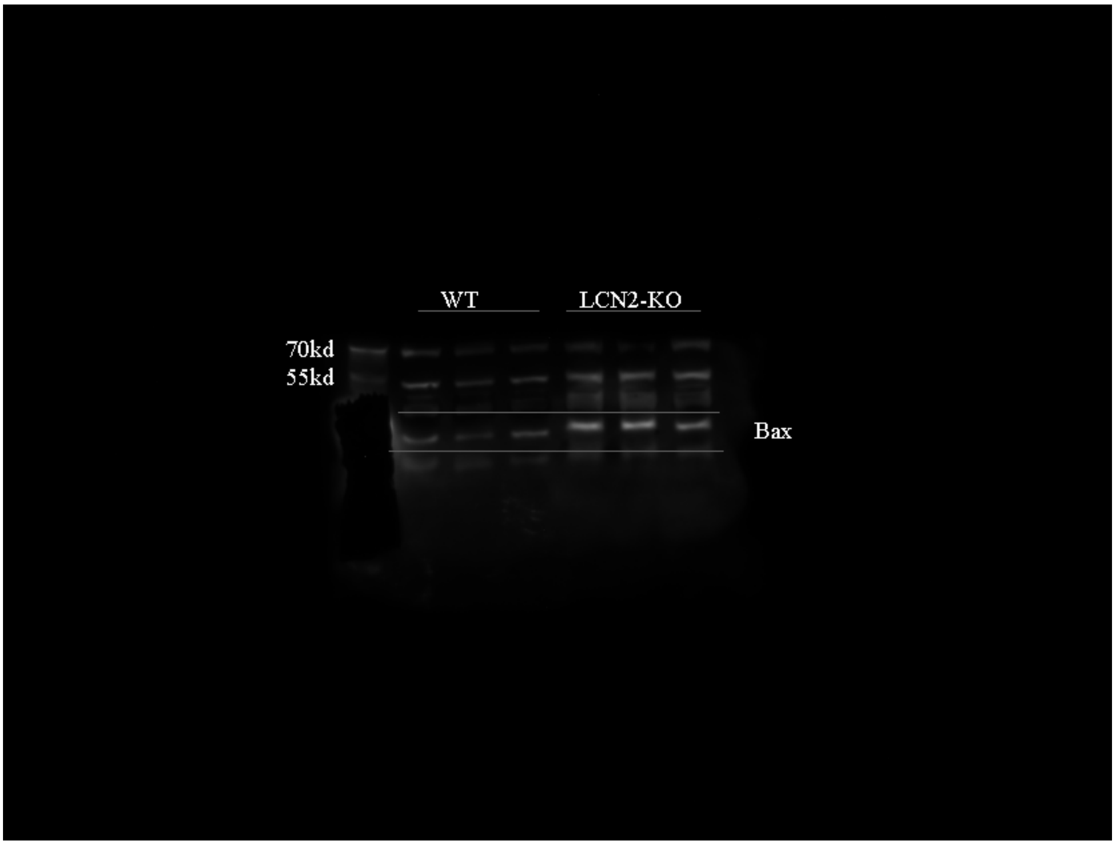

Figure S2 Full-length blot of Deptor protein expression level

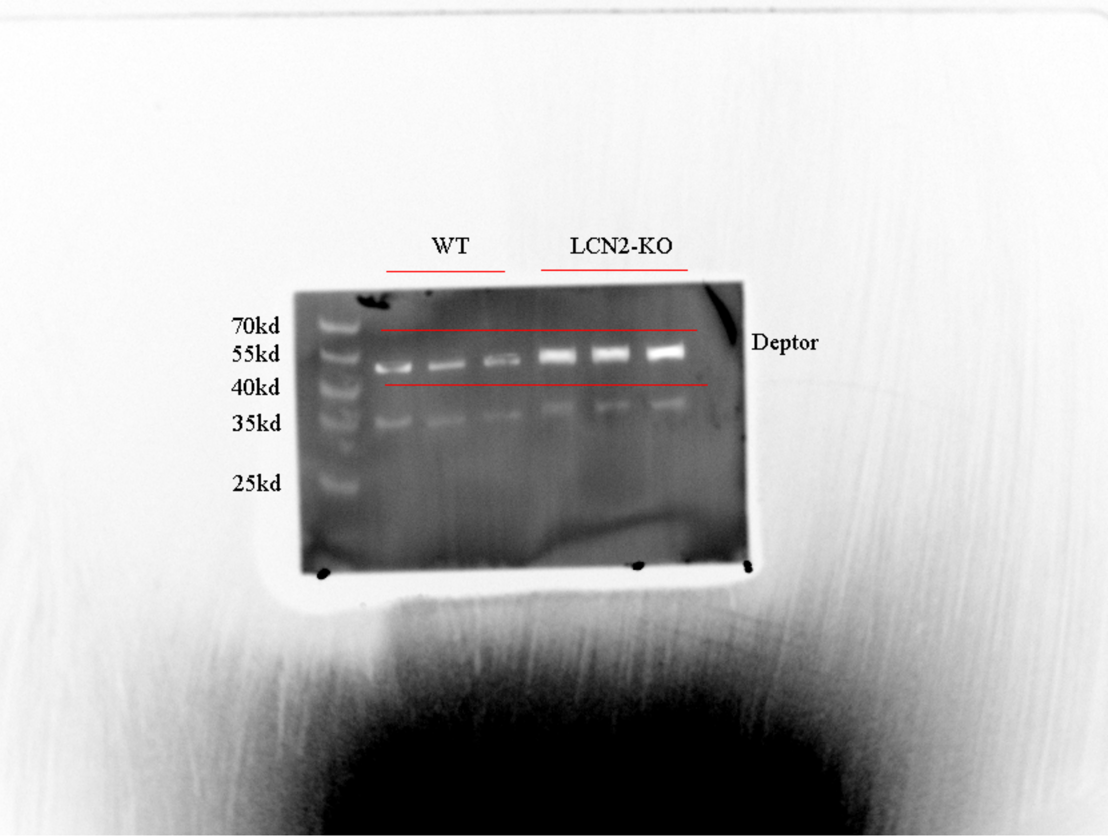

Figure S3 Full-length blot of β-tubulin protein expression level

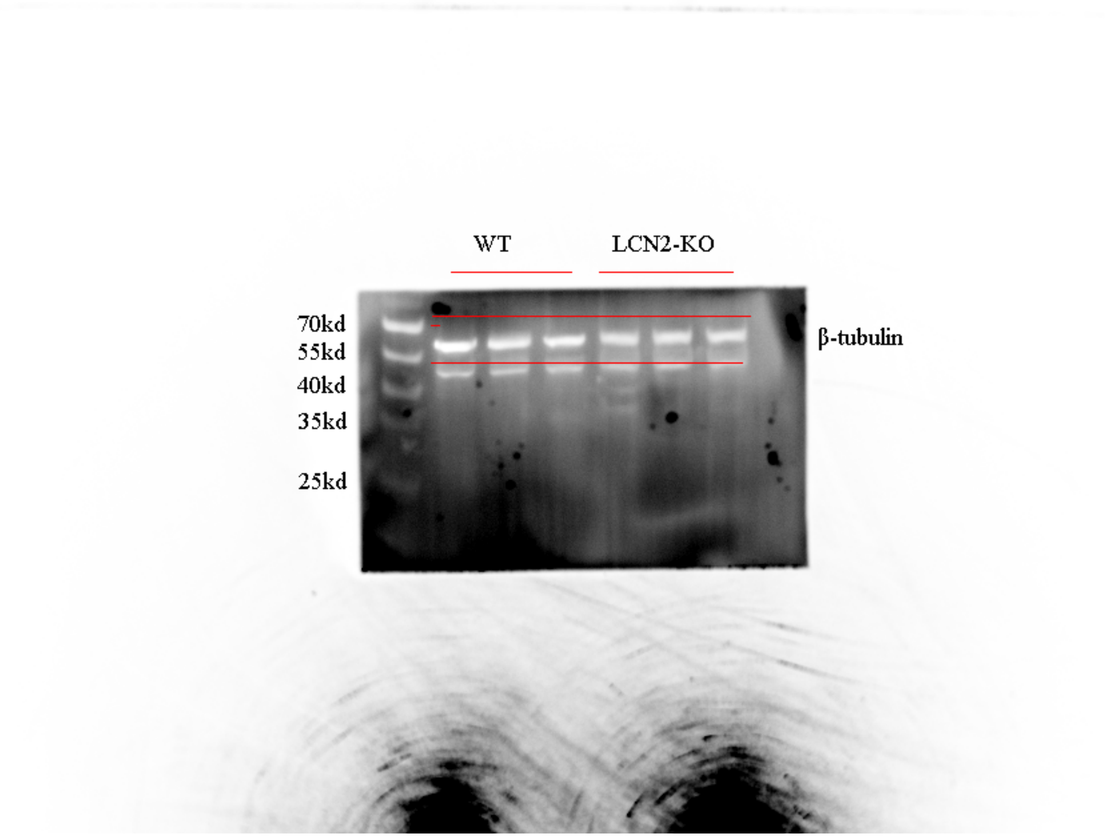

Figure S4 Full-length blot of Stat1 protein expression level

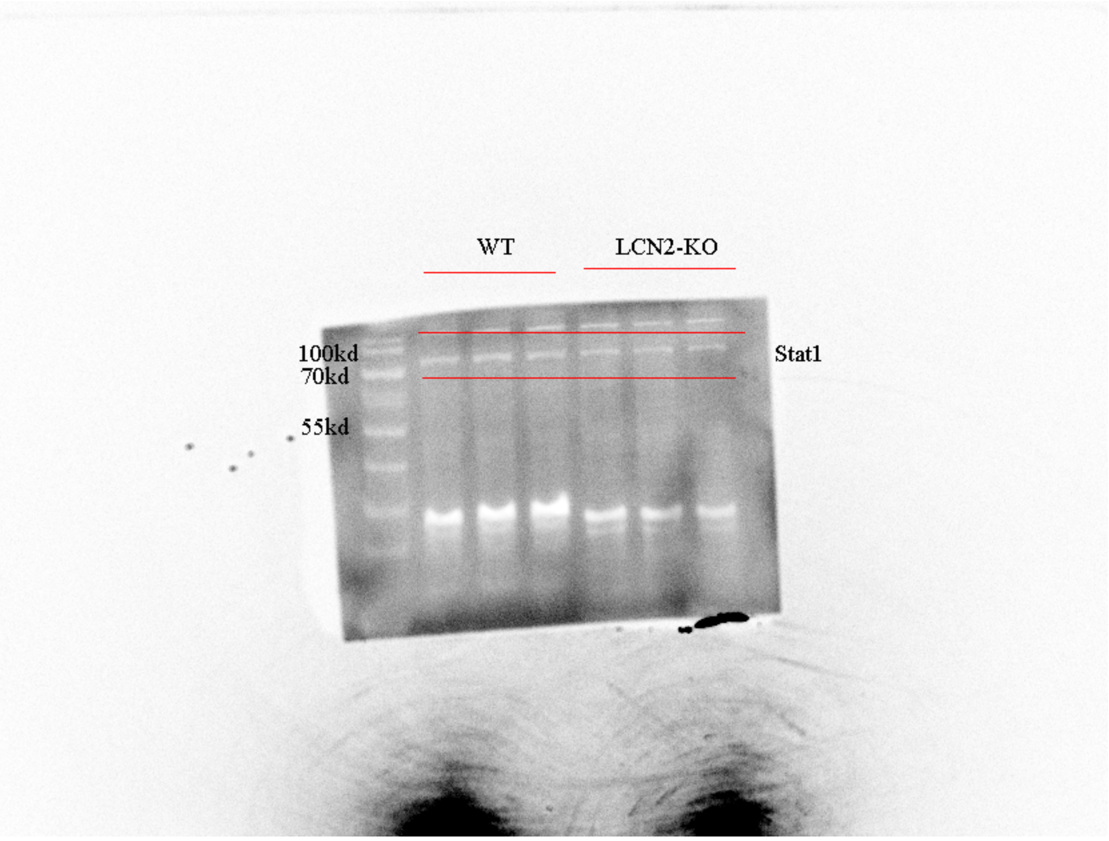

Figure S5 Full-length blot of β-actin protein expression level

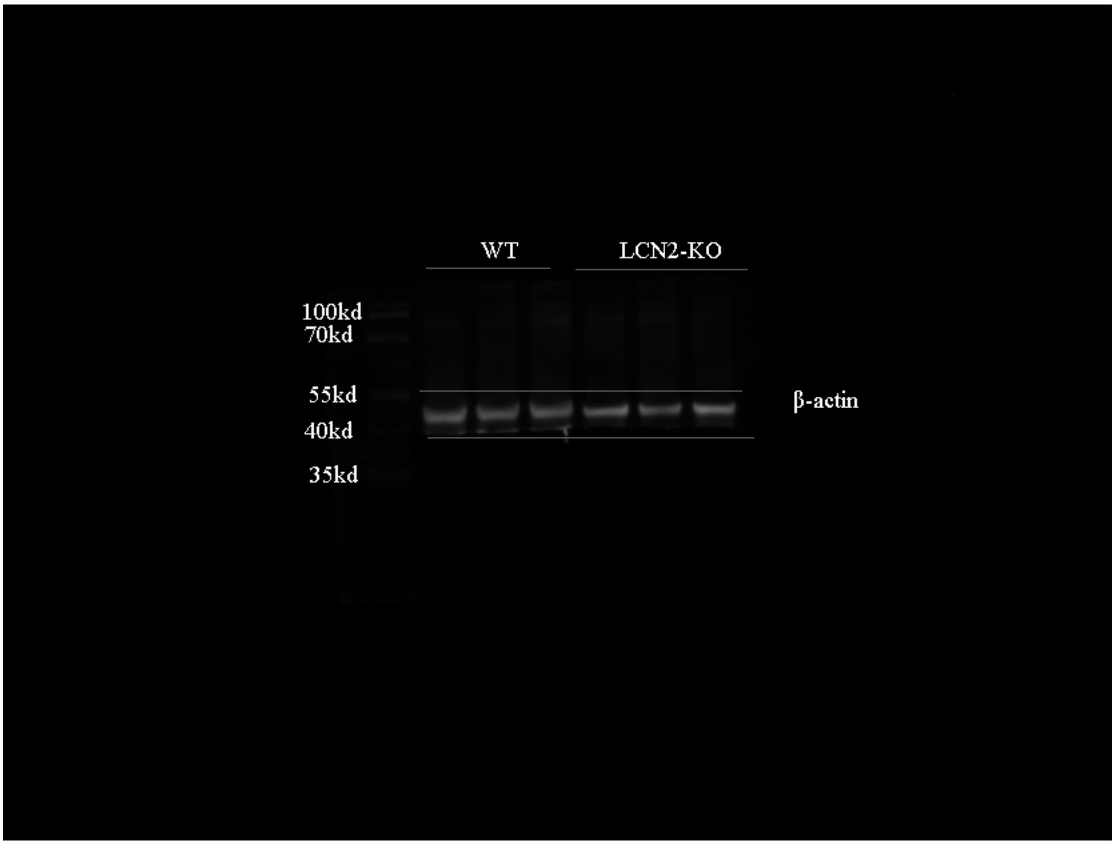

Figure S6 Full-length blot of Rps6ka1 protein expression level

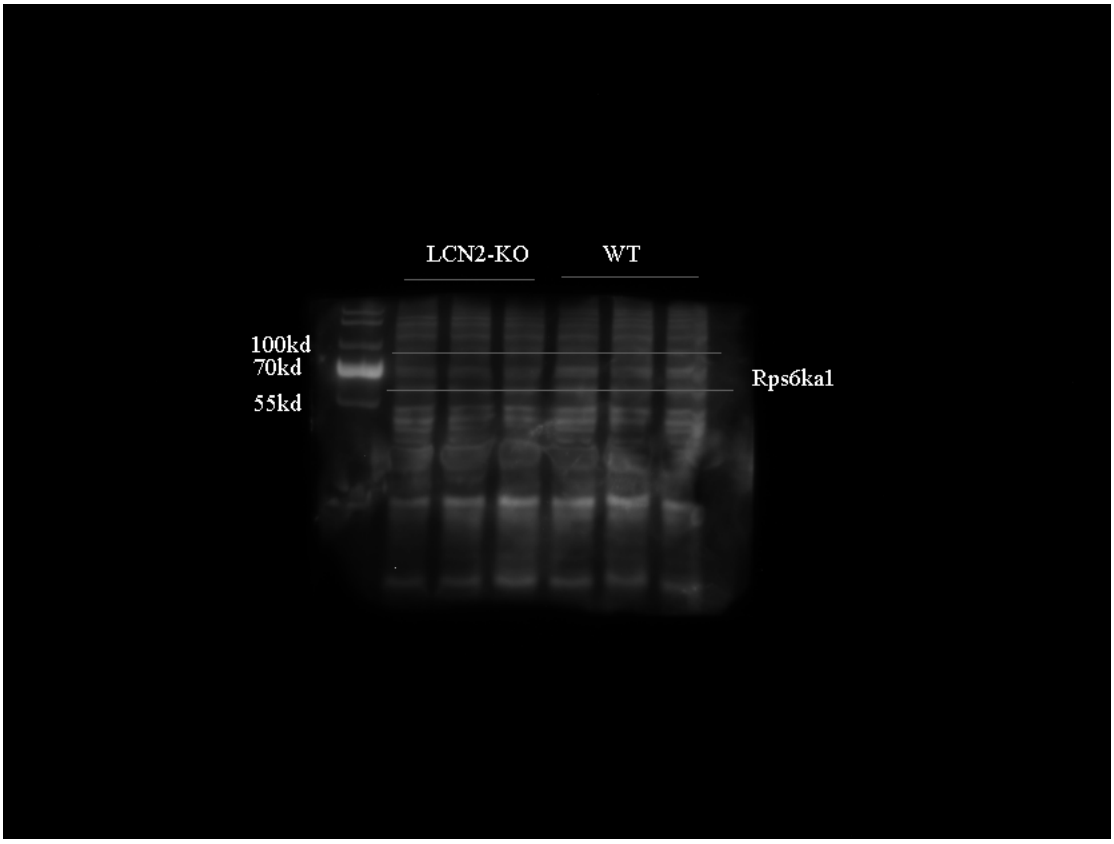

Figure S7 Full-length blot of GAPDH protein expression level

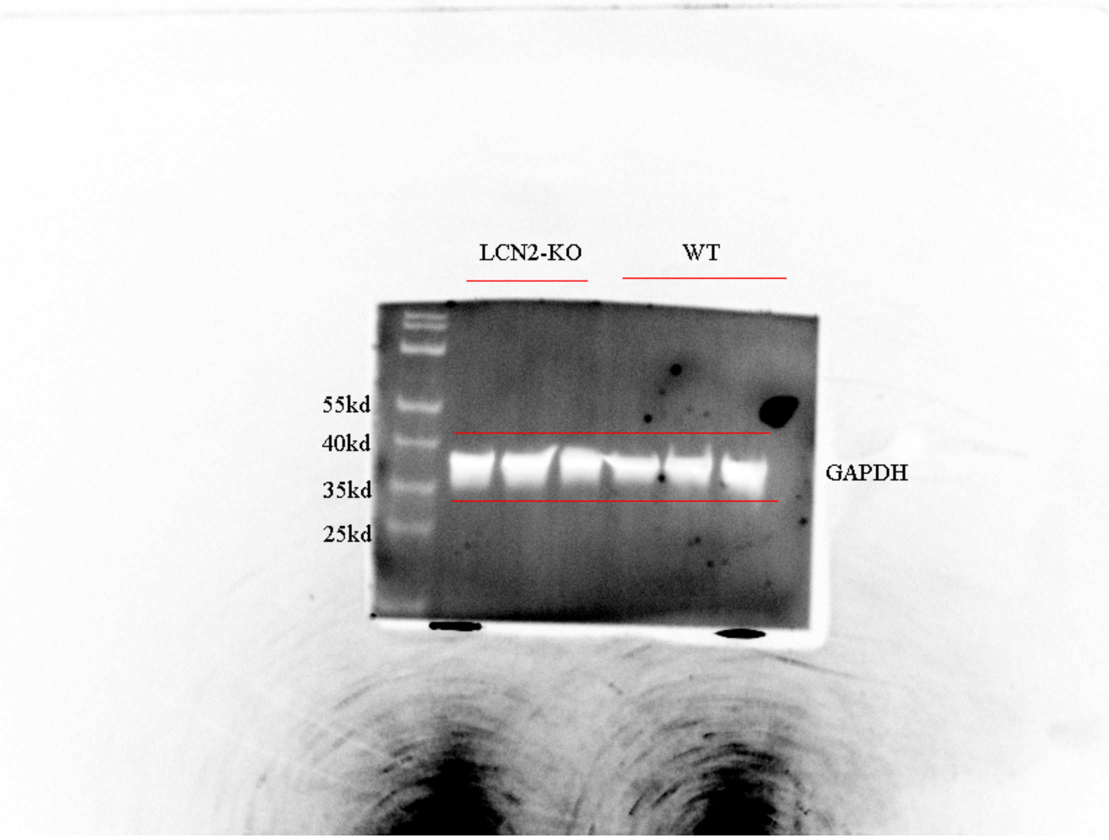

Supplement: Supplementary file 1 — Additional file 1: Figure S1. Full-length blot of Bax protein expression level. Figure. S2. Full-length blot of Deptor protein expression level. Figure. S3. Full length blot of β-tubulin protein expression level. Figure S4. Full-length blot of Stat1 protein expression level. Figure S5. Full-length blot β-actin protein expression level. Figure S6. Full-length blot of Rps6ka1 protein expression level. Figure S7. Full length blot GAPDH protein expression level. [file 12864_2021_8211_MOESM1_ESM.pdf]
